# An ecological study protocol for the multimodal investigation of the neurophysiological underpinnings of dyadic joint action

**DOI:** 10.3389/fnhum.2023.1305331

**Published:** 2023-12-06

**Authors:** Gabriella Tamburro, Patrique Fiedler, Antonio De Fano, Khadijeh Raeisi, Mohammad Khazaei, Lucia Vaquero, Ricardo Bruña, Hannes Oppermann, Maurizio Bertollo, Edson Filho, Filippo Zappasodi, Silvia Comani

**Affiliations:** ^1^Department of Neuroscience Imaging and Clinical Sciences, University “G. d’Annunzio” of Chieti–Pescara, Chieti, Italy; ^2^Behavioral Imaging and Neural Dynamics Center, University “G. d’Annunzio” of Chieti–Pescara, Chieti, Italy; ^3^Institute of Biomedical Engineering and Informatics, Technische Universität Ilmenau, Ilmenau, Germany; ^4^Center for Cognitive and Computational Neuroscience, Universidad Complutense de Madrid, Madrid, Spain; ^5^Department of Experimental Pschology, Cognitive Processes and Speech Therapy, Universidad Complutense de Madrid, Madrid, Spain; ^6^Department of Radiology, Universidad Complutense de Madrid, IdISSC, Madrid, Spain; ^7^Department of Medicine and Sciences of Aging, “University G. d’Annunzio” of Chieti–Pescara, Chieti, Italy; ^8^Wheelock College of Education and Human Development, Boston University, Boston, MA, United States

**Keywords:** multimodal experimental setup, dyadic motor task, synchronization, electroencephalography, kinematic data, table tennis, joint action

## Abstract

A novel multimodal experimental setup and dyadic study protocol were designed to investigate the neurophysiological underpinnings of joint action through the synchronous acquisition of EEG, ECG, EMG, respiration and kinematic data from two individuals engaged in ecologic and naturalistic cooperative and competitive joint actions involving face-to-face real-time and real-space coordinated full body movements. Such studies are still missing because of difficulties encountered in recording reliable neurophysiological signals during gross body movements, in synchronizing multiple devices, and in defining suitable study protocols. The multimodal experimental setup includes the synchronous recording of EEG, ECG, EMG, respiration and kinematic signals of both individuals via two EEG amplifiers and a motion capture system that are synchronized via a single-board microcomputer and custom Python scripts. EEG is recorded using new dry sports electrode caps. The novel study protocol is designed to best exploit the multimodal data acquisitions. Table tennis is the dyadic motor task: it allows naturalistic and face-to-face interpersonal interactions, free in-time and in-space full body movement coordination, cooperative and competitive joint actions, and two task difficulty levels to mimic changing external conditions. Recording conditions—including minimum table tennis rally duration, sampling rate of kinematic data, total duration of neurophysiological recordings—were defined according to the requirements of a multilevel analytical approach including a neural level (hyperbrain functional connectivity, Graph Theoretical measures and Microstate analysis), a cognitive-behavioral level (integrated analysis of neural and kinematic data), and a social level (extending Network Physiology to neurophysiological data recorded from two interacting individuals). Four practical tests for table tennis skills were defined to select the study population, permitting to skill-match the dyad members and to form two groups of higher and lower skilled dyads to explore the influence of skill level on joint action performance. Psychometric instruments are included to assess personality traits and support interpretation of results. Studying joint action with our proposed protocol can advance the understanding of the neurophysiological mechanisms sustaining daily life joint actions and could help defining systems to predict cooperative or competitive behaviors before being overtly expressed, particularly useful in real-life contexts where social behavior is a main feature.

## 1 Introduction

The life of every human being is permeated by spontaneous or deliberate, conscious or unconscious inter-individual interactions. These interactions can be accidental, e.g., when two people are crossing a narrow passage trying to avoid each other (Knoblich et al., 2011; Michael et al., 2020; Trendafilov et al., 2020; Ma et al., 2021), or intentional, e.g., when a couple is dancing a tango or medical doctors are performing surgery on a patient (Searle, 1990; Morales et al., 2019; Cassani et al., 2020). In the latter examples, the actions of the interacting individuals are interdependent and aim at achieving a shared goal, i.e., a goal that is common and cannot be achieved by acting independently from each other (Clark, 1996; Knoblich et al., 2011; Trendafilov et al., 2020; Ma et al., 2021). This type of interpersonal interaction is called joint action (Clark, 1996; Sebanz et al., 2006; Newman-Norlund et al., 2007).

Elucidating the mechanisms underpinning joint action may have an impact in a variety of social contexts involving mates, colleagues, competitors, genders, up to the understanding and improvement of decision-making mechanisms (Oullier and Basso, 2010; Krueger and Meyer-Lindenberg, 2019). The comprehension of the neurophysiological underpinnings of joint action may affect the development of best practices in the medical field (Leong et al., 2021; Short et al., 2021; Sened et al., 2022; Tucek et al., 2022) and in school teaching (Bevilacqua et al., 2019), it may help optimizing leadership processes or employees’ cooperativeness in corporate life (Astolfi et al., 2015; Zhang et al., 2019; Balconi et al., 2020; Boukarras et al., 2022), or it may support the improvement of sport performance (Filho et al., 2016; Cassani et al., 2020; Filho and Tenenbaum, 2020; Gugnowska et al., 2022).

Knoblich and Jordan (2002) were the first to hypothesize that human joint actions should be sustained by the activity of specific brain structures. They paved the way to the first neuroscientific studies on joint action, adopting the traditional stand-alone perspective of cognitive neuroscience investigating individual brains and bodies in (pseudo)social contexts (Newman-Norlund et al., 2007; Hari and Kujala, 2009; Sebanz and Knoblich, 2021; van der Wel et al., 2021). More recently, neuroscientists interested in joint action started to adopt a multi-person approach, where the unit of analysis is composed of the interacting individuals rather than considering each individual separately. Coordinated social interaction, especially that of real-time cooperative tasks, was then correlated with synchronized neural responses (Balconi and Vanutelli, 2017; Bolt and Loehr, 2021; Holroyd, 2022; Müller and Lindenberger, 2022). Interpersonal neural synchrony is a dynamic process in which the rapid changes occurring in the brain activity of one individual in association with a specific social behavior are perceived by the interacting partner and determine the changes occurring in his/her brain activity and behavior (Hamilton, 2020). From a methodological perspective, the study of brain-to-brain coupling underpinning joint action was permitted by hyperscanning investigations.

Hyperscanning, or hyperbrain scanning, is a neuroimaging approach consisting in the synchronization of multiple neuroimaging devices to investigate the synchrony of the neural signals and the brain-to-brain functional connectivity of two or more interacting individuals. It permits elucidating neural mechanisms of social interaction and identifying the neural networks and electrophysiological biomarkers associated with cooperative and competitive behaviors (Balconi and Vanutelli, 2017; Czeszumski et al., 2020; Nam et al., 2020). However, most real-life joint actions imply not only in-time synchronization of brain responses but also in-space coordination of body movements and face-to-face interactions. Recently, it has become clear that cognitive-motor processes and the neuropsychological mechanisms underpinning joint action should be investigated in ecological environments where individuals are free to move and interact (Redcay and Schilbach, 2019; Sebanz and Knoblich, 2021).

Ecological studies on group dynamics permitted to explore how being face-to-face could influence interpersonal relationships and brain-to-brain synchrony (Filho et al., 2016; Dikker et al., 2017; Ikeda et al., 2017; Bevilacqua et al., 2019; Stone et al., 2019). The mechanisms of interpersonal synchronization were mainly investigated by focusing on just the neural activations during cognitive paradigms involving only small movements (Babiloni et al., 2006; Novembre et al., 2017; Pan et al., 2017), or by assessing physiological and kinematic aspects (D’Ausilio et al., 2012; Chang et al., 2017) or specific psycho-physiological responses such as breathing and heart rate (Müller et al., 2021). However, a comprehensive investigation of joint action should not be limited to specific aspects or controlled situations. In fact, joint action derives from—and is sustained by—not only interpersonal neural synchrony, but also the coordinated physiological responses of other organ systems including the muscles, the heart, and the lungs (Filho et al., 2015, 2016, 2017; Müller et al., 2018; Coutinho et al., 2021; Mayo et al., 2021; Müller and Lindenberger, 2022).

From a systems perspective, interpersonal dynamics—hence joint action—is the ensemble of complex processes that are not only temporally linked across scales of analysis (e.g., short vs. long timescale), but also physically and informationally coupled across levels of analysis: neural (brain dynamics), cognitive-behavioral (action, dynamics of body parts), and social (dynamics of the behavioral coordination) (Gorman et al., 2017; Tognoli et al., 2018). To understand how interpersonal interactions are reflected on different levels of analysis, joint action should be studied with a more comprehensive multi-person approach that goes beyond hyperbrain scanning and integrates the assessment of neural signal synchrony and between-brain functional connectivity with the simultaneous monitoring of physiological responses (i.e., muscle activation, cardiac activity, and respiration), movement kinematics, and psychological states. A manipulation of joint action difficulty should be also considered to investigate how the complex system composed of the network of organs of two interacting individuals dynamically adjusts to variable external conditions (Rocca and Cavallo, 2018; Nordbeck et al., 2019). By doing so, both overt and covert processes occurring during interpersonal coordinated actions can be analyzed.

Yet, multi-person and multimodal neurophysiological studies focusing on joint actions that occur face-to-face, involve real-time and real-space coordinated movements, and consider both cooperative and competitive conditions are still missing (Czeszumski et al., 2020). This dearth of ecological, multi-person, and multimodal research on joint action can be attributed to different limitations: (1) technical limitations of the devices available to monitor hyperbrain activity during the performance of full-body movements; (2) lack of standards in the synchronization of multiple neurophysiological and kinematic imaging devices; and (3) shortage of study protocols suitable to investigate the underpinnings of joint action at multiple levels of analysis and to elucidate the influence of changing environmental conditions on the neurophysiological responses.

Although various neural imaging techniques have been used for hyperscanning studies, electroencephalography (EEG) is the most suitable to monitor brain activity during joint action because of its excellent time resolution (1 ms), high space resolution [from 32 up to 256 electrodes (Stoyell et al., 2021; Fiedler et al., 2022)], and portability. However, the challenge nowadays is to develop mobile EEG systems that are also wireless, mount electrodes suitable to monitor the brain during body motion, guarantee good electric contact while maintaining high wearing comfort for unobtrusive long-term applications. To study joint action with a multi-person and multimodal approach, it is also necessary to develop reliable technological solutions to synchronize the EEG systems with other devices able to monitor electromyographic, cardiac, respiratory, and kinematic signals. Finally, a suitable study protocol must be designed to fully exploit the above-mentioned technological advancements.

The aim of this article is to propose a solution for the synchronous acquisition of neurophysiological signals and kinematic data from two individuals involved in joint actions performed in both cooperative and competitive modes, and within an ecologic and naturalistic environment. We describe the experimental setup for dyadic multimodal acquisitions, which includes a novel class of electrodes specifically designed to monitor brain activity during the performance of full-body movements, and a solution to synchronize the devices to monitor brain activity, cardiac activity, respiration effort, muscle activation, and body movements from two interacting individuals. Finally, we describe how we designed a new study protocol that exploits the multimodal experimental setup to study the neuro-psycho-physiological underpinnings of joint action during the performance of an ecological full-body motor task that can be performed in both cooperative and competitive mode and at different difficulty levels. Our hope is that this proposal will help elucidating the influence of changing environmental conditions on the neurophysiological responses to cooperative and competitive joint action and will permit a multilevel and multimodal analysis of the collected data.

## 2 Methods and analysis

Our proposed protocol to synchronously acquire multiple neuro-physiological signals, kinematic data, and psychological traits from two interacting individuals includes three complementary parts: the experimental setup, the hardware and sensor components, and the procedures to record and analyze the multimodal data.

### 2.1 Experimental setup for ecological dyadic multimodal joint action studies

As described in the introduction, joint action studies have mainly focused on the synchrony of the neural signals and the between-brain functional connectivity of two or more individuals with stationary experimental protocols or with volunteers performing only small movements (for a review see, e.g., Czeszumski et al., 2020). Recently, researchers have integrated the monitoring of the neural function with that of the physiological activity of other organ systems, such as the heart, the lungs, and the epidermis. This integration can provide useful information to investigate the responses of the autonomous nervous system to interpersonal coordination (e.g., Toppi et al., 2016; Vanutelli et al., 2018; Cassani et al., 2020; Coutinho et al., 2021; Sciaraffa et al., 2021; Lange et al., 2022). However, these studies generally did not extend beyond monitoring the activity of two organ systems due to the technological difficulties encountered in synchronizing multiple devices. So far, there is no study on joint action where multiple physiological signals are recorded synchronously with kinematic data and psychological states from multiple interacting individuals during the performance of a full-body motor task (Czeszumski et al., 2020).

**FIGURE 1:**
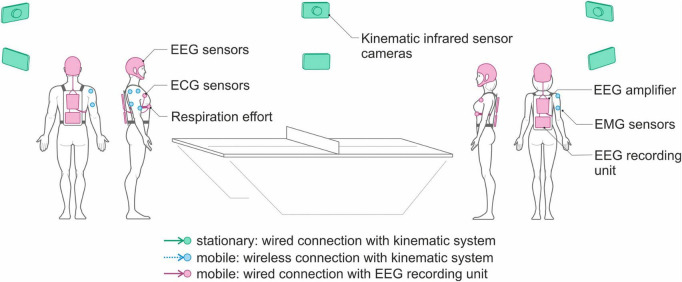
Schematic representation of the experimental setup comprising two mobile EEG devices and one stationary motion capture system.

Our experimental setup was conceived to record neural, myographic, cardiac, respiratory and kinematic data from two individuals performing an ecological joint action involving full body movements and face-to-face interpersonal interactions. To this aim, we employed two mobile EEG systems (eego sports EE-225, ANT Neuro B.V., Hengelo, Netherlands)—one for each volunteer—for recording EEG, electrocardiographic (ECG) and respiratory signals, and one stationary motion capture system (BTS SMART-DX, model DX6000) with an integrated wireless electromyographic (EMG) system for recording kinematic and muscle activity data from the two interacting individuals. A schematic representation of our experimental setup is given in Figure 1.

Each mobile EEG system is composed of:

1. An electrode cap, mounting 64 novel dry electrodes specifically developed to record brain activity during the performance of free body movements, as described in the following subsection.
2. A mobile biosignal amplifier (64 referential channels for EEG plus two bipolar inputs for ECG and Respiration acquisitions) recording at a sampling rate of 1024 Hz.
3. A respiration belt using a piezo-electric sensor.
4. Bipolar self-adhesive hydrogel electrodes for monitoring the ECG.
5. A tablet computer for multimodal data recording and storing (i.e., EEG, ECG, and respiration).

The two EEG systems are mobile, which means that the eego amplifier, the data recorder, and a sensor input box for ECG and respiration effort are placed inside a backpack worn by the individuals participating in the study, whereas signal recording is controlled remotely.

When preparing the volunteers for the dyadic multimodal acquisitions, the respiration belt is positioned around the chest at the base of the sternum, whereas the bipolar electrodes for ECG monitoring are positioned at the fourth intercostal space, symmetrically on the sides of the thorax (Rajaganeshan et al., 2008). The EEG, ECG and respiration signals from the two players are then synchronously recorded by the mobile EEG system at a sampling frequency of 1024 Hz. The motion capture system consists of 8 infrared cameras connected to a PC for the acquisition of the kinematic data using infrared-reflective markers. Movement kinematics are recorded at 250 frames per second. Additionally, two video cameras are also connected to the acquisition PC to record analog videos that can be used in the post-processing phase.

**FIGURE 2:**
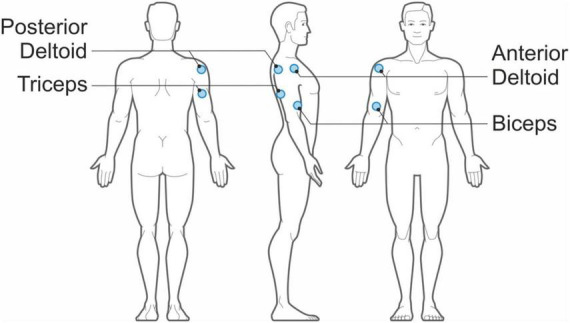
Positions of the bipolar EMG sensors on each table tennis player.

The EMG signals from the two participants are acquired using the EMG system integrated within the motion capture system. Given that eight wireless bipolar EMG sensors are available, for each volunteer we can record the electrical activity of four muscles of the upper right or left arm, according to the handedness of the volunteer. Figure 2 shows the location of the EMG sensors, positioned on the anterior deltoid, posterior deltoid, biceps, and triceps.

#### 2.1.1 New electrodes for monitoring brain activity during full body movements

State-of-the-art techniques supporting mobile, unobtrusive neuroimaging include functional near-infrared spectroscopy (fNIRS) and EEG. Accordingly, synchronization of two or more simultaneous recordings allows studying interacting individuals (Sänger et al., 2011; Lee et al., 2019; Czeszumski et al., 2020; Müller et al., 2021). Both modalities are portable and provide a high temporal (fNIRS > 10 ms; EEG > 1 ms) and spatial resolutions (fNIRS > 20 mm; EEG > 5 mm). However, on both aspects, state-of-the-art mobile EEG can be considered superior to mobile fNIRS systems. Most specifically, commercial mobile HD-EEG systems comprise, e.g., 64 to 256 lightweight electrodes (Delaux et al., 2021; Stoyell et al., 2021; Fiedler et al., 2022, 2023) fully integrated into cap systems covering the entire scalp. Therefore, EEG may currently be the best choice for hyperscanning studies of joint actions involving full body movements.

Nonetheless, presently available commercial mobile EEG systems have several limitations: (1) wireless systems often comprise very limited channel counts, specifically for peripheral sensors like ECG, EMG, and respiration; (2) multimodal setups often are too bulky or heavy for full body movements; (3) wiring (e.g., for sensors), weight, and size of the devices limit body movements, and thus negatively affect the ecological recording conditions intended; (4) sensors and cables are highly susceptible to motion artifacts; (5) caps usually provide low wearing comfort; (6) system application is complex, time-consuming, and error-prone—requiring specifically trained operators; and (7) limited recording sessions per day are possible due to setup complexity and cap cleaning and drying.

Recent advances in hardware development for mobile EEG and mobile brain/body imaging have made great strides addressing these limitations, including the implementation of (1) compact amplifier modules for HD-EEG with 64 and up to 128 channels; (2) electronic concepts for reduction of environmental noise and movement related artifacts (e.g., active electrodes or active shielding) (Xu et al., 2017; Kappel et al., 2019); (3) protocols and tools for wireless data and event transmission; and (4) novel dry electrode technologies, with unique characteristics allowing fast electrode preparation and cleaning, no risks of short-circuits due to gel running between adjacent electrodes, and no signal degradation due to sweat.

Dry electrodes are the most suitable type of electrode to study neural activations with high spatial resolution and low preparation time in ecological protocols involving full body movements. The most commonly used commercial mobile EEG systems supporting dry electrodes include waveguard touch by ANT neuro (Fiedler et al., 2015, 2022; di Fronso et al., 2019); g.SAHARA and unicorn by g.tec (Pontifex and Coffman, 2023); epoc and insight by emotive (Duvinage et al., 2013); Drytrode by neuroelectrics; actiCap Xpress by Brain Products (Mathewson et al., 2017); Drypad and Flex by Cognionics (Mullen et al., 2015). However, the electrodes in these systems are either rigid or offer very low flexibility, thus exhibiting low adaptivity to individual head shapes and increased impedances, requiring specific electronics (Fiedler et al., 2015, 2018, 2022), and limited wearing comfort (Vasconcelos et al., 2021; Ng et al., 2022).

Based on this analysis of existing systems and technologies, we derived the following requirements for the development of novel dry electrodes and cap systems for studying dyadic multimodal joint action: (a) improved electrode flexibility, shape, and softness for high wearing comfort; (b) improved cap design for a tight fit and a high level of adjustability to individual head sizes and shapes; (c) maintained signal quality and channel reliability; (d) reduced susceptibility to movement artifacts; and (e) compatibility with state-of-the-art mobile amplifiers supporting multimodal data acquisition.

**FIGURE 3:**
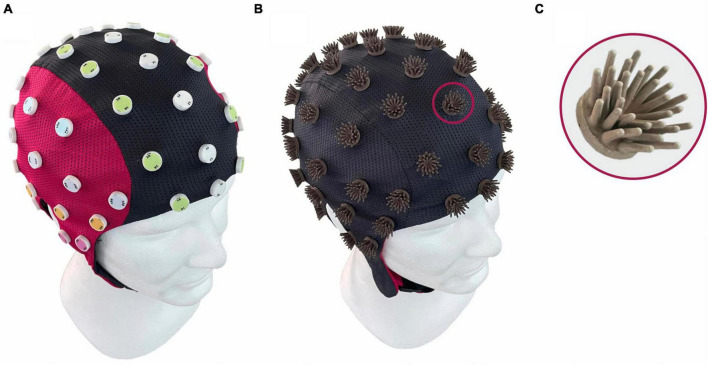
Design of the novel Flower electrode and cap: **(A)** cap from the outside showing the equidistant electrode layout (di Fronso et al., 2019) and **(B)** turned inside out showing the individual flower electrodes; **(C)** magnification of a single flower electrode with 30 pins (Warsito et al., 2023).

To this aim, we developed a novel dry electrode (Warsito et al., 2023), specifically addressing the needs of ecological joint action studies. The novel design is based on our previous dry electrodes (Fiedler et al., 2015, 2022; di Fronso et al., 2019) using an electrically non-conductive but mechanically flexible polyurethane substrate. A subsequent coating of Silver/Silver-Chloride (Ag/AgCl) is applied using a multi-phase plating process (Mota et al., 2013), which ensures reliable signal quality (Fiedler et al., 2014, 2015), electrochemical stability, and mechanical durability (Fiedler et al., 2022). The novel flower-shaped design and specifically adapted substrate hardness (Fiedler et al., 2018) ensure both a reliable electrode-skin contact and considerably increased comfort (Warsito et al., 2023). The individual angled pins, arranged in an intertwined layout on a common baseplate, allow pre-defined deformation of the electrode and therefore adaptation to individual head shapes and compensation of excessive pressure during application and wearing. Within a flexible fabric cap, the electrodes are arranged in an equidistant layout both for reasons of optimal global pressure distribution and mechanical adduction as well as to facilitate later signal analysis using connectivity metrics on sensor and source level. Figure 3 shows a photography of the 64-channel cap with an equidistant electrode layout.

#### 2.1.2 Synchronization solution for dyadic multimodal joint action studies

No system allowing the synchronized recording with high sampling rates of dry HD-EEG, ECG, EMG, respiratory and kinematic data for dyadic multimodal joint action studies is currently commercially available. Consequently, reliable technological solutions able to synchronize multiple measurement modalities, and thus devices from different vendors, necessary for the simultaneous monitoring of the neurophysiological underpinnings of joint action, are still missing.

Moreover, no common international standard for the synchronization of multiple (mobile) devices from different vendors has been implemented so far. Consequently, the synchronization must be achieved using a solution specifically developed for the given setup, allowing the individual recordings of different systems to be aligned after the acquisition, enabling subsequent joint analysis. Three different approaches for *post hoc* data alignment can be distinguished, in order of increasing accuracy:

1. Start/stop alignment: It requires a common event to be sent to/shared with all modalities, indicating the start and/or the end of a given recording interval. This approach requires high accuracy of the event time stamps and assumes equal transmission time of the event to all receiving modalities, as well as homogeneous sampling within each modality during the recording. Start/stop alignment is most often implemented using technical signals like TTL trigger events shared with all modalities.
2. Constant alignment: It is an extension of the start/stop alignment that uses periodic events sent to all modalities and recorded together with each individual system. The periodic events allow for compensating drifts in the clock (thus in the sampling rate of the individual modalities), therefore increasing the accuracy of the *post hoc* data alignment.
3. Shared channel alignment: This approach makes use of an individual signal (i.e., a channel) being redundantly recorded by all modalities. This redundant signal is used for *post hoc* data alignment. The signal may be of biological (e.g., EMG, accelerometer) or technical origin. Although very high accuracy is theoretically possible to achieve in the *post hoc* data alignment, the realistic accuracy is highly dependent on the equal quality and low noise in the common signal recorded at all modalities. Differences in the sensor position, sampling rate, and noise level will cause differences in the signals, increasing the uncertainty and reducing the quality of the *post hoc* data alignment.

A practical setup, therefore, is to use common events shared between all modalities. Conventional events based on TTL triggers are suitable for stationary modalities and require a cable running to each device, rendering them inapplicable for mobile setups. A related quasi-standard for cross-vendor and cross-modality synchronization of data in research applications exists: the Lab Streaming Layer (LSL) middleware system (Kothe, 2014; Wang et al., 2023). LSL uses a local area network infrastructure (WLAN or ethernet) and allows both data and event streams to be shared online during an ongoing recording. Given the network-based infrastructure, LSL can be used both in wired and wireless setups. Although LSL is an open-source networking middleware, not all vendors or manufacturers support LSL for their products, and the existing vendor’s implementations often show considerable differences and limitations in stream format timing (i.e., time stamp), accuracy, temporal resolution, and signal latency (Iwama et al., 2022).

Our technological setup, therefore, requires a dedicated specific arrangement fulfilling the following core functions: (a) as few devices and as low complexity as possible; (b) support for wireless data and event distribution; (c) support for central monitoring and control; (d) data alignment across multiple devices/modalities, independent from temporal resolution (sampling rates); (e) continuous periodic event distribution to allow compensation of sampling inconsistencies; and (f) high event timing accuracy.

**FIGURE 4:**
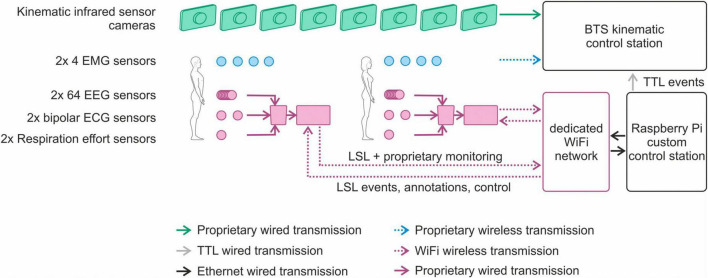
Overview of the novel synchronization solution for control, monitoring and *post hoc* data alignment in multimodal joint action studies.

We thus developed and implemented a novel synchronization solution enabling the joint control, monitoring, and *post hoc* data alignment of the multiple individual devices for multimodal joint action studies with the requirements described and defined above. For this purpose, we implemented a centralized control unit running on a real-time Linux operating system (Raspbian GNU/Linux 10) on a Raspberry Pi (version 4, model B, Raspberry Pi Foundation, Cambridge, UK). The central control unit is connected to the stationary control unit of the kinematic monitoring system via individual cables between the digital input/output interface pins of the control unit and the trigger event ports of the kinematic system. Moreover, the central control unit is connected via a dedicated wireless network to the mobile EEG recording units carried by each volunteer. An overview of the resulting setup is shown in Figure 4.

The status of the individual devices and sensors can be monitored online, using their respective proprietary control software. In addition, the status of the multiple mobile recording units can be monitored using the eego web server interface (version 1.9.3, ANT Neuro B.V., Hengelo, Netherlands) running on the central control unit.

**FIGURE 5:**
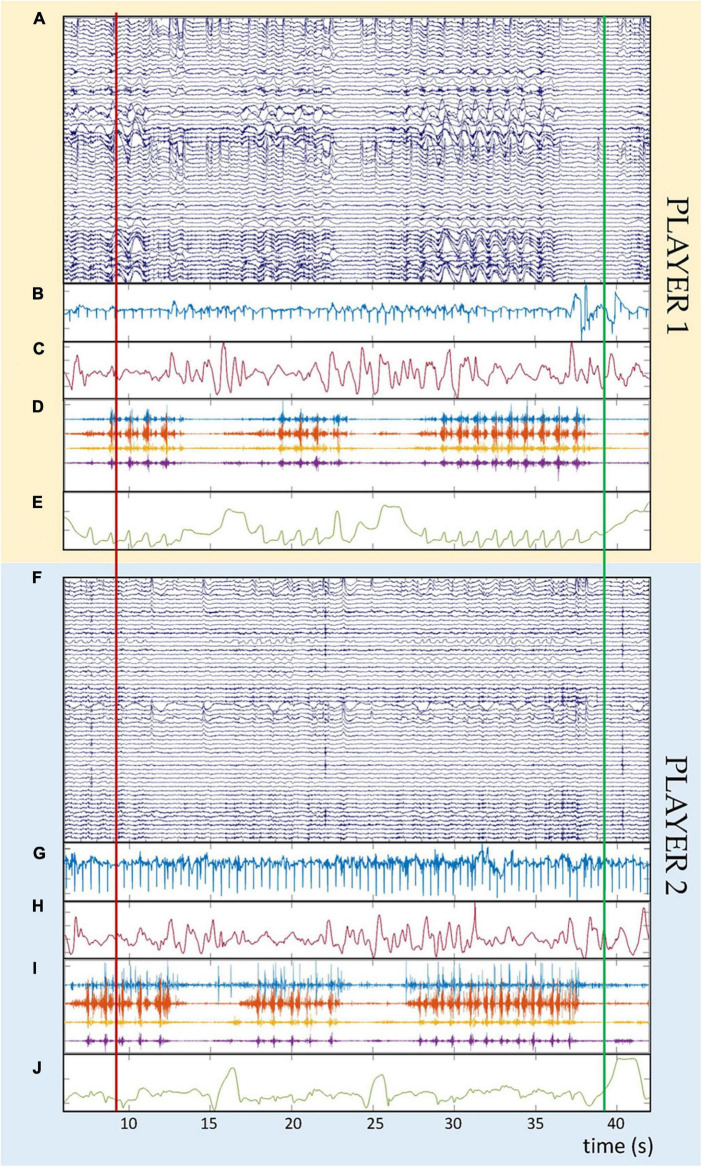
Example of synchronous multimodal physiological data recorded in one dyad during cooperative table tennis. Panels **(A–E)** refer to one member of the dyad (player 1), whereas panels **(F–J)** refer to the second member of the dyad (player 2). Panels **(A,F)** show the raw dry EEG recordings (64 channels, amplitude in μV). Panels **(B,G)** show the cardiac signals (one channel, amplitude in mV). Panels **(C,H)** show the respiration effort (one channel, amplitude in%). Panels **(D,I)** show the electromyographic signals recorded from four muscles per player (four channels, amplitude in mV). Panels **(E,J)** show the absolute value of the position vector of the marker placed on top of the table tennis racket (one trace, amplitude in m). The time scale at the bottom of the figure is identical for all panels and both players. The vertical red and green lines over all panels indicate the start and stop event triggers used for synchronization purposes.

Via individual scripts running in a Python environment (version 3.7, Python Software Foundation, DE, USA) and executed from the control unit, events (i.e., recording start, recording stop, and continuous periodic synchronization) can be sent quasi-simultaneously to the kinematic monitoring system (via TTL triggers) and to the mobile recording units (via LSL events sent via the dedicated wireless network). Moreover, the operator can add individual annotations to the recordings either via the web interface or dedicated Python scripts. The individual events are recorded in sync with the respective sensor signals and can be used for *post hoc* data alignment. Figure 5 shows an example of multimodal data synchronously recorded from two members of a dyad. Recordings include multichannel dry EEG, ECG, respiration effort, EMG and kinematics of the racket.

### 2.2 Design of the new study protocol for ecological dyadic multimodal joint action studies

The design of a study protocol for investigating dyadic joint action with an ecological and multimodal approach requires to address several requirements: (a) the choice of the dyadic task; (b) the definition of the conditions to realize different task difficulty levels; (c) the identification of the requirements for a multilevel analysis of the multimodal data; and, from a non-technical perspective, and (d) the definition of a method to select the study population.

#### 2.2.1 Choice of the dyadic task

To fulfill the condition of an ecological study protocol, it is important to identify a dyadic task that can be performed by replicating natural body movements during the interpersonal interaction (i.e., task analysis). Moreover, the joint action task should be characterized by face-to-face interactions and should be suitable for both cooperative and competitive joint actions to permit identifying specific functional features that characterize—and differentiate—the neurophysiological activations during these different types of interaction. Finally, to investigate how the complex interpersonal dynamics sustaining joint action change when the external conditions are modified, it is necessary that the task can be performed at various difficulty levels during both cooperation and competition.

Expanding from our previous paradigm for studying cooperative juggling (Filho et al., 2015), we identified table tennis as a dyadic motor task suitable to implement an ecological joint action study where the participants freely move and interact with each other and with the environment. Moreover, table tennis entails real-time, real space, and face-to-face interactions between the individuals, involves natural full-body movements, and permits to implement both cooperative and competitive joint actions. In fact, during the cooperation condition, the players can dribble as they do before a match, whereas during the competition condition they can play table tennis like in a match. Finally, table tennis is suitable to be played at various difficulty levels by implementing modifications in the playing conditions, as described in the next section.

#### 2.2.2 Conditions to realize different task difficulty levels

To study the influence of changing external conditions on the neurophysiological activations underpinning joint action, a minimum of two different task difficulty levels should be implemented, i.e., an easy and a hard level.

The easy level is based on the normal playing environment, where the players can use the whole table during both cooperative and competitive conditions. On the other hand, a suitable hard level must fulfill two requirements: (1) the volunteers must feel that playing table tennis is noticeably more difficult than at the easy difficulty level; and (2) the table tennis rallies (i.e., the intervals during which table tennis is actually played, which start with the initial stroke of a player and end when the ball falls off the table) must have—on average—a duration comparable with that of the rallies played at the easy level, in order to permit the functional analysis of the neurophysiological data.

The hard level can be implemented by adopting various strategies, such as modifying the height of the net or the weight of the ball. We chose to partially mask the surface of the table tennis table. The concept is that the masked portions of the table cannot be used for playing: if the ball falls on the prohibited areas, the rally is stopped. This condition introduces a strong disturbance in the standard playing interaction. To define the portions of the table that must be masked to obtain an optimal hard difficulty level, both the percentage of the covered table area and the shape of the mask must be defined, as both can have an influence on the perception of the players that the match is more difficult.

To identify the conditions that best realize the hard difficulty level for both cooperation and competition, we recruited 5 dyads of volunteers. Given that the players’ skill level can influence their perception of game difficulty, they were selected according to the procedure described in section “2.2.4 Selection of the study population” below. The dyads played multiple trials of table tennis (each lasting 1 min) using various mask types. To assess the effectiveness of each mask, the volunteers rated their perceived effort by assessing a modified Borg scale after each trial (Borg and Borg, 2010; Staiano et al., 2018). The Borg scale consists of 11 items describing the level of perceived effort which ranges from 0 (extremely easy) to 10 (extremely hard).

**FIGURE 6:**
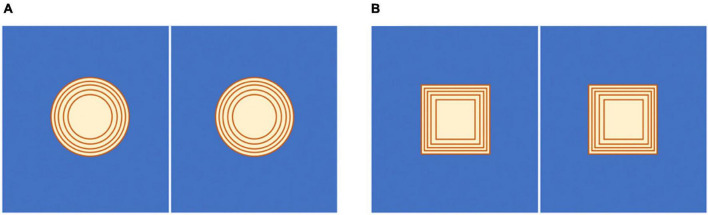
Schematic representation of the masks that have been tested to identify the ideal hard difficulty level. **(A)** Area reduction with a circle mask. **(B)** Area reduction with a square mask. The area of the table is reduced by 10, 15, 20, 25, and 30%, starting from the smallest internal mask to the largest external one in both pictures. The players can use only the blue area of the table tennis table.

We first tested a mask that simply reduced the table tennis dimensions (both width and length) by increasing percentages up to 50%. However, all players reported no perceived increase in task difficulty with this type of mask. Therefore, we evaluated two other masks, one with the shape of a circle and one with the shape of a square, which were placed at the center of each half of the table. The dimension of the masks varied to achieve five different percentages of table area reduction: 10, 15, 20, 25, and 30% (see Figure 6).

Each dyad played a total of 22 1-min trials, each corresponding to a different combination of playing condition (cooperation or competition), difficulty level (easy or hard), mask shape (circle or square), and percentage of table reduction (10, 15, 20, 25, or 30%). The trials were performed in randomized order.

At the end of the tests, we calculated the distribution of the perceived effort, as reported by the volunteers after each trial. Then, we used a two-tailed non-parametric Wilcoxon signed rank test to explore for differences between the various hard difficulty levels (for the two mask shapes) with respect to the easy difficulty level. A variant of the Cohen’s d related to non-parametric tests was used to calculate the effect size for significant differences (Fritz et al., 2012; Tomczak and Tomczak, 2014). Given the importance of the rally duration for the subsequent functional analysis of the neurophysiological data, we also calculated the mean rally duration across dyads for the easy difficulty level and for each combination of mask shape and percentage of table reduction. The rally durations were calculated by analyzing the kinematic data collected with the motion capture system.

**FIGURE 7:**
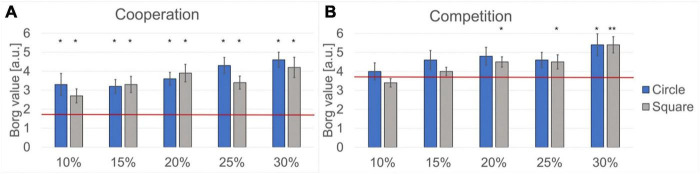
Analysis of the reported Borg scale values. The horizontal red lines indicate the mean Borg scale values for the easy difficulty level. The height of the columns indicates the mean Borg scale value for the hard difficulty level, and the error bars indicate the Standard Error Mean (SEM). Significant differences between the easy and the hard difficulty levels are marked (*p < 0.05 and **p < 0.005). **(A)** Results obtained during cooperation. **(B)** Results obtained during competition.

Figure 7 shows the average Borg scale values reported by the volunteers for the different hard difficulty levels (i.e., combinations of circle/square mask and percentage of table reduction) for both cooperation and competition. For comparison, the average Borg scale value reported for the easy cooperation and easy competition levels is indicated with a horizontal red line.

Regarding the perceived effort (Borg scale), significant differences between the easy and the hard difficulty levels during cooperation were observed for both masks and for all percentages of table area reduction. The more significant differences (*p* < 0.005, *r* > 0.5) were observed for both masks at 20, 25, and 30% of table area reduction.

Significant differences between the easy and the hard difficulty levels during competition were observed for the square mask at 20% (*p* < 0.005, *r* = 0.535), 25% (*p* < 0.05, *r* = 0.485), and 30% (*p* < 0.005, *r* = 0.580) of table area reduction. For the circle mask we observed significant differences only at 30% of table area reduction (*p* < 0.05, *r* = 0.491).

**FIGURE 8:**
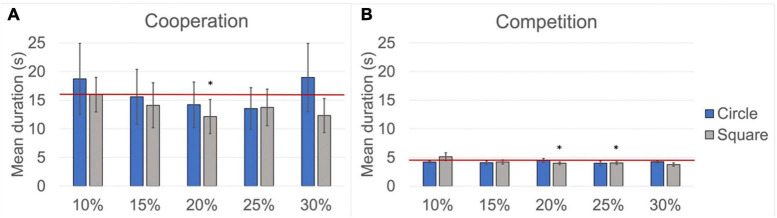
Mean rally duration. The red lines indicate the mean rally duration for the easy difficulty level. The height of the columns indicates the mean rally duration for the hard difficulty level, and the error bars indicate the Standard Error Mean (SEM). Significant differences between the easy and the hard difficulty levels are marked (*p < 0.05). **(A)** Results obtained during cooperation. **(B)** Results obtained during competition.

Figure 8 provides an overview of the mean rally durations for the different difficulty levels for both cooperation and competition. For comparison, the average rally duration during the easy cooperation and easy competition levels is indicated with a horizontal red line.

Regarding the average rally duration, a significant difference between the easy and the hard difficulty levels during cooperation was observed only for the square mask at 20% of table area reduction (*p* < 0.05, *r* = 0.450), whereas significant differences during competition were observed only for the square mask at 20% (*p* < 0.05, *r* = 0.379) and 25% (*p* < 0.05, *r* = 0.147) of table area reduction.

From the results reported above, we observed that, for competition (which is the most critical playing condition), the square mask at 30% of table area reduction achieves the most significant statistical difference for the perceived effort between the easy and the hard levels while maintaining a comparable average rally duration between the two difficulty levels. Therefore, this mask fulfills the requirements to implement a suitable hard difficulty level and was chosen for our proposed study protocol.

#### 2.2.3 Definition of the requirements for the multilevel analysis of the multimodal data

**FIGURE 9:**
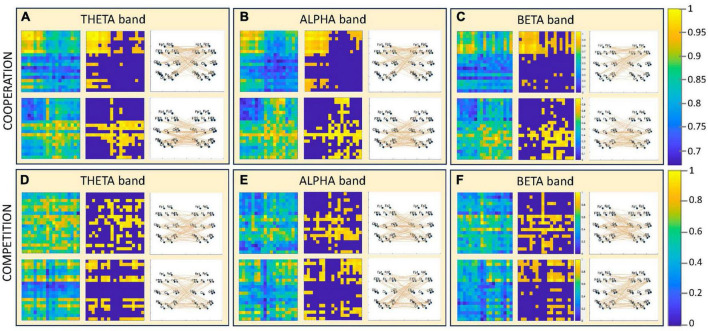
Examples of hyperbrain functional connectivity maps during the performance of cooperative and competitive table tennis. The maps are obtained from one dyad and from subsets of 19 channels representative of all brain areas (one subset for each member of the dyad). Each panel **(A– F)** refers to a unique combination of condition (cooperation or competition) and frequency band in which the EEG data were analyzed (theta, alpha, and beta bands). Each panel includes two rows, each referring to one of the two dominant hyperbrain functional connectivity maps observable for that specific combination of condition and frequency band. In each row the functional connectivity maps are shown, from left to right, as phase synchronization maps [calculated with the Phase Lag Index (PLI)], as adjacency matrices (where only the 30% of the highest functional connections are retained), and as dominant interbrain functional connections. At the right-hand side of the figure, there are the color scales for the maps: the one at the top refers to the PLI maps, whereas the one at the bottom refers to the adjacency matrices.

The wealth of information contained in the multimodal data that can be recorded with our experimental setup warrants the definition of adequate analytical methods. The neural level of analysis includes the assessment of hyperbrain functional connectivity, the evaluation of the topological properties and integration/segregation balance of the functional maps by means of Graph Theoretical measures (Büchel et al., 2021), and the estimation of the global brain dynamics using a microstate analysis approach (Michel and Koenig, 2018). For example, hyperbrain functional connectivity during cooperation and competition can be estimated using various algorithms, such as the phase locking index (PLI), or algorithms employing the imaginary part of the phase locking value (PLV), which are less sensitive to volume conduction problems (Bruña et al., 2018). Figure 9 shows examples of dominant hyperbrain functional connectivity maps for a dyad during the performance of cooperative and competitive table tennis. The maps are calculated in three frequency bands: theta (4–8 Hz), alpha (8–12 Hz), and beta (12–30 Hz). The balance between integration and segregation of the functional maps can then be measured through global/local efficiency algorithms, and the global hyperbrain dynamics can be assessed on hyperbrain microstate maps from input EEG signals built using the hyperbrain recordings. Finally, adjusted microstate features (Croce et al., 2022) can be used to characterize the hyperbrain microstate maps and dynamics, such as the mean joint microstate duration and the mean joint occurrence.

From our perspective, however, a multilevel analytical approach should be preferred, where the neural level of analysis is integrated with the information extracted from the kinematic data to achieve a cognitive-behavioral level of analysis through group analysis of the hyperbrain results obtained before, during, and after each table tennis rally. Kinematic information such as the speed of the ball exchange (that varies between cooperation and competition but also across dyads) or the distance between the table tennis rackets can be used to see how the features of the cortical patterns change as a function of different kinematic conditions. This approach permits to identify specific patterns of hyperbrain activations for the cooperative and competitive conditions and to explore how they change as a function of an external perturbation, achieved by changing the difficulty level of the task.

Finally, a more comprehensive view of the mechanisms underpinning joint action can be reached by integrating the neural and cognitive-behavioral analyses with a functional connectivity analysis that involves all the recorded neurophysiological signals. Within a Network Physiology approach, the analysis of the dynamic interactions among the diverse physiological organs activated during joint action (i.e., brain, muscles, heart, lungs) can be applied to a system composed of two interacting human beings. Given that the fundamental frequencies of the functional dynamics of different organ systems (brain, heart, muscles, and lungs) are not comparable, the corresponding neurophysiological signals cannot be simply pooled together and processed by means of a functional connectivity approach. Our proposed solution is to use a windowing and feature extraction strategy that can be adopted to generate proxy timeseries with the same number of elements for the same time intervals. The elements of each timeseries will be pertinent features of the neurophysiological signals, such as connectivity features or power spectral density (PSD) for brain activity, muscle activation onset for the EMG, heart rate variability for the ECG and respiration rate for respiration effort. To represent the statistical dependencies between the various physiological organs, a functional network can be built using the feature timeseries extracted from the neurophysiological signals. This proxy functional network can then be analyzed by means of [e.g., Graph theoretical measures such as degree (to find the nodes with central role in the connectivity network), betweenness centrality (to find the nodes that act as bridges between different communities or modules in the network), clustering coefficient (to evaluate clustering or interconnectedness among a node’s neighbors), modularity (to find functionally related physiological processes), and local and global efficiency (to evaluate whether the functional connections are more local or expanded across the brain)]. Performed at both individual and dyadic levels, inter-organ functional connectivity patterns can be integrated with kinematic information and the results of psychological tests to reach a social level of analysis. This approach permits to explore how diverse physiological systems in the human organism dynamically interact and collectively behave to produce distinct physiologic states and functions (Bartsch et al., 2015).

However, the methods available to calculate functional connectivity require a minimum duration of the neural signal to provide reliable results, which is typically indicated as being 500 ms (Basti et al., 2022). We should then consider that—for the planned neural analysis—the intervals of interest are the table tennis rallies, which can be as short as 1 or 2 s, especially during the competition condition (see Figure 8). Furthermore, the segmentation of the EEG recordings into intervals of interest can introduce signal distortions at the edges of the EEG intervals, requiring the exclusion of part of the signal at the beginning and end of each interval. This approach, although necessary, further reduces the duration of the intervals of interest that can be used for the planned analysis. It is therefore necessary to determine the minimum rally duration that is sufficient to ensure reliable functional connectivity results.

At the cognitive-behavioral analysis level, the interpersonal interactions are characterized by kinematic features, such as the relative phase between players, that can be employed to segment the neural signals and perform group analysis of the individual and hyperbrain functional connectivity maps during the different table tennis phases. Please note that there is a trade-off between spatial and temporal resolution in motion capture systems, with a higher frame rate (i.e., temporal resolution) allowing only the recording of less detailed images (i.e., spatial resolution). Therefore, it is important to choose a sampling rate for acquiring kinematic data that represents a time resolution sufficiently high to achieve a reliable segmentation of the neural signals but low enough to maintain good image resolution for the reconstruction of movement kinematics.

Finally, we should keep in mind that the multiple neurophysiological signals cannot be simply pooled together and processed to implement the social level of analysis. In fact, their intrinsic time scales are highly diverse: in a healthy adult at rest, brain dynamics (as measured by a standard clinical EEG) occur in a frequency range of 1–40 Hz (Tatum, 2014; Capilla et al., 2022), activity related to muscular contraction is typically included in the range between 20 and 40 Hz (Shue and Brozovich, 1999), the average heart rate ranges from 1 to 1.6 Hz, and the average respiratory rate is between 0.2 and 0.33 Hz. One solution would be resampling all signals to a lower sampling rate, but this process may lead to signal distortion and loss of information. To avoid these issues, a windowing and feature extraction strategy can be adopted to generate feature timeseries with the same sampling rate from the various neurophysiological signals. The sampling rate can be adjusted through window length and overlap. By doing so, all feature timeseries can be compared and analyzed according to a functional connectivity approach across a common time scale. However, this approach implies that the neurophysiological recordings must be sufficiently long to ensure the reconstruction of suitable feature timeseries.

The neurophysiological signals and kinematic data recorded during the test acquisitions performed to define the hard difficulty level were also used to (1) determine the minimum duration of rallies to be retained for subsequent analysis, (2) assess the suitability of the chosen sampling rate for kinematic data acquisition, and (3) set a total duration of the recordings that can be suitable for the social level of analysis.

The rally duration can be defined by tracking the position of the ball and table tennis rackets. In fact, two motion capture markers are placed on each racket, one on the top and one on a side, to distinguish its left and right sides, whereas the table tennis ball is coated with the same reflective material of the markers, being visible by the infrared cameras. The same company that produces the markers for the motion capture system coated the table tennis balls. The weight of a coated ball, that in normal conditions is around 3 g, is increased by about 1 g. Although the weight of a coated ball is higher of that of an uncoated ball, the players who participated in our tests did not report any perceived effect of the ball coating on their play.

**FIGURE 10:**
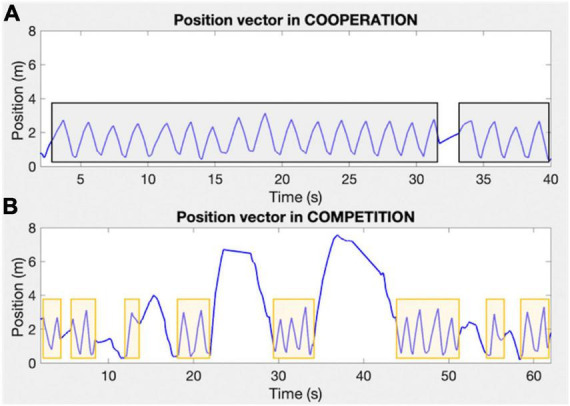
Examples of vectors expressing the position of the coated ball during cooperative and competitive table tennis [**(A,B)** panels, respectively]. The *X*-axis expresses the time in seconds, whereas the *Y*-axis expresses the ball position in meters. The gray and orange rectangles indicate the table tennis rallies during cooperation and competition, respectively.

Therefore, it is possible to reconstruct the instants—within a rally—when the ball is hit by the racket or when it falls off the table. By identifying these key instants, we can identify the individual rallies within a table tennis match and reconstruct the kinematic variables necessary for the subsequent analysis of the neurophysiological signals (i.e., the sub-periods of individual exchanges within each rally). Figure 10 shows an example of ball tracking during a table tennis rally performed in cooperative (upper panel) and competitive (lower panel) modes.

We then analyzed the duration of each rally during the competition condition played at the hard difficulty levels because it leads—on average—to the shortest rallies (see Figure 8). This analysis, in combination with that of the video-recordings, permitted to verify that rallies of 3 s duration are sufficiently long to guarantee that at least 2 table tennis exchanges between the players are recorded. This minimum number of exchanges is crucial to have rallies representative of joint action. Furthermore, rallies lasting at least 3 s ensure sufficiently long recordings after having removed the initial and final 500 ms. Therefore, we concluded that the minimum duration of the rallies to be retained for further analysis should be 3 s, and that rallies shorter than 3 s should be disregarded.

We also confirmed that a sampling rate of 250 Hz for the kinematic data permitted a reliable segmentation of the neurophysiological signals (acquired with a sampling rate of 1024 Hz). Although kinematic data could be acquired at higher sampling frequencies (up to 1 kHz), 250 Hz permits a reasonable computation time to reconstruct movement kinematics while keeping a high image resolution (2.8 Megapixel).

We further verified that the total duration of the table tennis sessions should not exceed 60 min. In fact, the volunteers generally reported to be very tired after 1 h of continuous playing. For this reason, our acquisition protocol is composed of multiple sessions of 2.5 min duration each, and the volunteers can take breaks between subsequent sessions to rest and compensate for fatigue. Furthermore, given that several rallies during competition can be shorter than 3 s and therefore excluded from data analysis, we decided to record, for each difficulty level, 4 sessions for cooperation and 8 sessions for competition. By doing so, we ensure the acquisition of neurophysiological recordings lasting 10 (20) minutes for cooperation (competition) at each task difficulty level, which is considered sufficient for the social level of analysis.

#### 2.2.4 Selection of the study population

Another aspect to be considered when defining the study protocol is that long neurophysiological recordings might induce fatigue effects in the players, altering their performance. We have already considered these effects by limiting the total duration of an acquisition and allowing for breaks between playing sessions. However, fatigue effects may prevent poorly skilled volunteers to complete the sessions, whereas too highly skilled volunteers could perform too many rallies that are too short to be retained for data analysis. Therefore, the ideal volunteers for our study should have prior experience in table tennis but should not be professionals (i.e., should not be enrolled in a national or regional table tennis federation).

Assessing the skill level of the volunteers has further advantages: (1) the members of a dyad can be skill-matched, hence avoiding remarkable skill differences that might introduce a bias in the neurophysiological activations and therefore alter the results of the statistical group analysis; (2) the dyads can be split in two groups of higher and lower skilled volunteers to allow studying whether different neurophysiological activations occur as a function of skill level, as demonstrated in a previous study on cooperative juggling (Stone et al., 2019); and (3) encompassing a range of players that spans from less skilled to more skilled, our study population becomes more representative of the general population, whose mechanisms underpinning joint action are our ultimate research focus.

**FIGURE 11:**
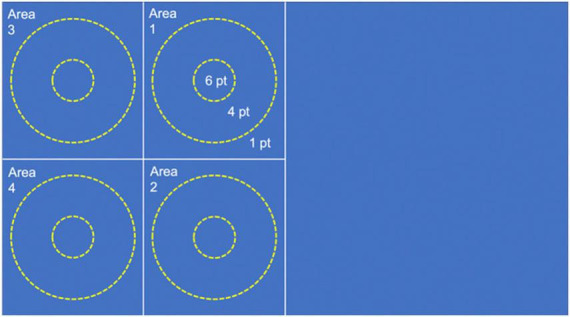
Representation of the targets positioned on one half of the table tennis table to test the service skill.

Based on these premises, we defined a battery of 4 practical tests to assess the level of abilities needed to participate in our table tennis joint action study. These tests were designed to assess the technical proficiency necessary to effectively perform table tennis movements (i.e., forehand stroke, backhand stroke, forehand service and backhand service), and also permit to evaluate the perceptuo-motor skill for ball control and the eye-hand coordination skill. The four tests are as follows:

1. Test for forehand stroke skill: The volunteers perform consecutive forehand strokes against the opposite half of the table tennis table positioned vertically. They are instructed to make as many forehand strokes as possible in a time interval of 30 s. If the ball falls, the volunteer can pick up the ball and continue. The test ends when the time interval is over. The result is the highest number of consecutive forehand strokes.
2. Test for backhand stroke skill: The same as test 1, but volunteers perform consecutive backhand strokes instead of forehand strokes.
3. Test for forehand service skill: The volunteers perform forehand services by trying to place the ball in one of 4 quadrants in which the opposite half of the table tennis table is divided. Two concentric round targets are positioned at the center of each quadrant: one with a diameter of 60 cm and another one with a diameter of 20 cm (see Figure 11). Two attempts for each quadrant are allowed (i.e., 8 services in total). Each service is assigned a score, based on where the ball falls, similarly to the study by Faber et al. (2015): 0 points if the ball does not fall in the assigned quadrant; one point if the ball falls in the assigned quadrant but outside the round targets; four points if the ball falls in the assigned quadrant and inside the larger target; six points if the ball falls in the assigned quadrant and inside the smaller target. The result is the sum of the highest scores obtained for each quadrant.
4. Test for backhand service skill: The same as test 3, but volunteers perform backhand services instead of forehand services.

After having completed all tests, a total score is assigned to each volunteer, which is the sum of the scores obtained in the 4 tests. The members of each dyad are selected by matching their final scores.

These scores are also used to define two skill level groups for statistical group analysis of the neurophysiological results: the median of all volunteers’ scores is calculated and two skill level groups are defined: a less skilled group including the volunteers with a total score below the median, and a more skilled group including the volunteers with a total score above the median.

#### 2.2.5 Protocol for multimodal table tennis joint action studies

After having defined the dyadic task, the conditions to realize different task difficulty levels, the requirements related to the multilevel analysis of the multimodal data, and the tests to select the study population, the study protocol can be defined. It includes the recruitment phase and the study paradigm for the multimodal data acquisition.

Recruitment phase: Before participating in the study, the volunteers must complete some forms to provide demographic information and some questionnaires to check whether they satisfy the inclusion and exclusion criteria, listed in Table 1.

**TABLE 1:** List of inclusion and exclusion criteria.

Inclusion criteria	All volunteers MUST:
	1. Have no known neurological, psychiatric or systemic pathologies
	2. Be physically active (Global Physical Activity Questionnaire)
	3. Have previous experience in table tennis (Questionnaire)
	4. Be aged between 18 and 35 years
**Exclusion criteria**	**All volunteers MUST NOT:**
	1. Be in a physical or mental clinical condition that may compromise the normal physiological functioning, permanent or temporary
	2. Be sedentary
	3. Be part of or have been part of table tennis national federations in the past (e.g., Federazione Italiana Tennis tavolo)
	4. Have taken part in official table tennis tournaments
	5. Have previous and/or present gaming experience with one or more recruited study volunteers
	6. Have taken on alcohol or coffee in the 24 h preceding the start of the experiments
	7. Have taken any other drugs in the 7 days preceding the start of the experiments

If the volunteers satisfy the inclusion and exclusion criteria, they provide written informed consent, undergo the skill level assessment tests (see section “2.2.4 Selection of the study population”), and complete psychometric instruments that assess personality traits of extraversion/introversion, cooperativeness/competitiveness, individual social preferences or motivations (i.e., pro-social or pro-self), and shared and complementary mental models:

1. The Big Five Inventory test (John and Srivastava, 1999; Ubbiali et al., 2013).
2. The Cooperative/Competitive Personality Scale (CCPS) (Lu et al., 2013).
3. The Social Value Orientation Scale (SVO) (Murphy et al., 2011).
4. The Team Mental Models Instrument (TMM-I) (Filho et al., 2022).

The results of these tests can be used to help interpret the results of the social level analysis of the multimodal data.

Study paradigm for multimodal data acquisition: Before data acquisition, the dyads are composed by matching the members of each dyad by skill level and handedness. Also, the dyad members are administered the Psychobiosocial Scale (Robazza et al., 2016) to assess their functional/dysfunctional psycho-bio-social states. The results of this test will be used to interpret the results of the group analysis of the neurophysiological data. Then, the dry EEG caps, respiration belts, bipolar electrodes for ECG, motion capture markers, and EMG sensors are mounted on both volunteers according to the description given in the section “2.1 Experimental setup for ecological dyadic multimodal joint action studies.”

**FIGURE 12:**
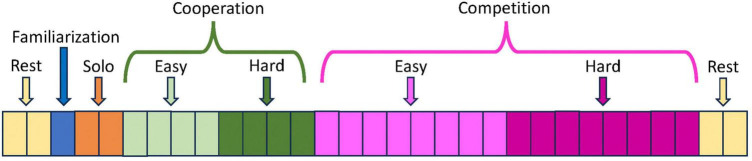
Structure of the study paradigm. Each rectangle in the bar represents an individual data acquisition session of 2.5 min duration. The cooperation and competition sessions played at easy and hard difficulty levels are executed in randomized order (not indicated in the figure).

The study paradigm is composed of several sessions according to the scheme given in Figure 12. No specific instructions are given to the volunteers, who are free to play table tennis as they usually do in both cooperation and competition, maintaining an ecological setting. After each session, the volunteers can take breaks. The sessions related to cooperation and competition are played at both easy and hard difficulty levels in randomized order.

During REST (6 min), all electrophysiological signals are acquired with eyes open, with the volunteers sitting in front of a wall and fixating a red cross on a white paper positioned at eye level and at 3 meters distance. This passive control condition aims to collect baseline EEG, ECG, and respiration data before and after the table tennis match, when the volunteers are not engaged in any specific task or interaction.

During FAMILIARIZATION (2.5 min), the members of the dyad can familiarize with each other in playing table tennis.

During SOLO (2 intervals of 2.5 min), all electrophysiological and kinematic signals are recorded for each member of the dyad while they individually play against the opposite half of the table tennis table positioned vertically. Volunteers are required to play as long as possible and with a constant high speed, trying to avoid errors. The purpose of this active control condition is to establish baseline data for the electrophysiological and kinematic signals during full-body movement, but without a social interaction component.

During COOPERATION (4 intervals of 2.5 min for each difficulty level), the volunteers try to keep the ball within the playing area for as long as possible. When the ball falls off the table, they restart immediately. Volunteers are instructed to alternate in the service.

During COMPETITION (8 intervals of 2.5 min for each difficulty level), each member of the dyad attempts to outperform his counterpart by scoring more points, as it usually happens during a standard competition. When the ball falls off the table, they quickly resume playing. Volunteers are instructed to alternate every two services, as in real competitions.

## 3 Discussion

In the last decade, ecological studies on group dynamics were performed to elucidate the neural mechanisms of cooperative and competitive social interaction (Balconi and Vanutelli, 2017; Czeszumski et al., 2020; Nam et al., 2020), and the influence of being face-to-face on brain-to-brain synchrony (Filho et al., 2016; Dikker et al., 2017; Ikeda et al., 2017; Bevilacqua et al., 2019; Stone et al., 2019). These studies employed cognitive paradigms with only small movements (Babiloni et al., 2006; Novembre et al., 2017; Pan et al., 2017), and the assessment of either physiological and kinematic aspects (D’Ausilio et al., 2012; Chang et al., 2017) or specific psycho-physiological responses (Filho et al., 2015, 2016, 2017; Müller et al., 2018, 2021; Coutinho et al., 2021; Mayo et al., 2021; Müller and Lindenberger, 2022).

To our knowledge, the multimodal experimental setup and study protocol proposed in this article are the first solutions designed for the synchronous acquisition of multiple neurophysiological signals together with kinematic data and personality traits from two individuals engaged in ecological and naturalistic joint actions, encompassing both cooperation and competition.

The novel experimental setup was specifically developed for this purpose, and allows recording EEG, ECG, EMG, respiratory, and kinematic data of dyads during full-body movements. The proposed setup has been successfully implemented and its function has been verified through preliminary test recordings. The reduction to two device types per volunteer (i.e., three devices overall, given that the motion capture system with integrated EMG is common to both volunteers), each connected to multiple sensors, in combination with the novel sensor types including the new dry EEG electrodes, has contributed to significantly reducing the preparation effort and time, dropping the overall setup complexity, and ensuring ecological, naturalistic recording conditions. Moreover, our novel solution for the inter-device synchronization has been technically validated and implemented in the selected devices, facilitating *post hoc* data alignment and joint data analysis.

Regarding hardware aspects, as a result from our planned study, we expect further insight on required optimization such as minimization of the devices and recording/control units; weight and cabling reduction; ease and reliability of the sensor preparation and application; and EEG electrode and cap reliability. This input will ultimately lead to a revision of the current setup, facilitating ecological studies not only in the chosen application of table tennis, but possibly in a wide range of similar protocols for the study of interpersonal interaction. The chosen approach for the inter-device synchronization can be integrated into the software of each device for further reduction of setup complexity and easy extension to additional devices for other studies and paradigms.

An important aspect in the definition of the proposed study protocol regarded the selection of the dyadic task. This selection was guided by the need to identify a motor task that permitted to realize a naturalistic interpersonal interaction in a laboratory setting. Table tennis was chosen due to its faithful replication of real-game conditions in our experiments, featuring face-to-face interactions and unconstrained interpersonal coordination in time and space. Additionally, table tennis has the advantage over other dyadic motor tasks that it is suitable to be played in both cooperative and competitive mode. This is a mandatory requirement to understand how the neurophysiological underpinnings of joint action change in real life during these different conditions of interpersonal interaction without introducing biases stemming from dissimilar tasks undertaken in cooperative and competitive contexts. Finally, table tennis is appropriate to implement two difficulty levels that, by mimicking changing external conditions, permit studying how these occurrences might influence the neurophysiological mechanisms sustaining joint action.

During the initial test recordings, we verified that the sampling rate chosen for acquiring kinematic data was adequate for a reliable segmentation of the neurophysiological signals and also defined the minimum rally duration for a reliable functional connectivity analysis of the neural signals. As well, we defined the overall duration of the neurophysiological data acquisitions to prevent the occurrence of fatigue effects in the volunteers while enabling the reconstruction of sufficiently long proxy data series to implement a Network Physiology approach for the analysis of the neurophysiological signals.

Notably, we also carefully considered the type of volunteers to be included in our study, intentionally excluding professional or semi-professional table tennis players because they are not representative of the general population. By assessing the skill level of the volunteers with the defined practical tests, we not only cover a wide range of skill levels in table tennis, but also obtain that the members of each dyad can be skill-matched, hence facilitating the categorization of the dyads into a higher and lower skill level groups aimed at exploring the impact of skill level on the neurophysiological activations underpinning joint action.

It is worth mentioning that our protocol includes the recording of not only joint action sessions but also resting state and individual table tennis sessions, which may provide useful insights on how neural activity patterns differ between interactive (joint action) and non-interactive conditions. Resting state recordings allow establishing the baseline neural activity of each individual when they are not engaged in any specific task or interaction. By comparing this baseline with the neural activations occurring during joint action, we may better identify the brain regions and neural networks involved in social interactions. Additionally, individual table tennis sessions (the SOLO condition in our protocol) permit to identify the neural activations occurring during individual motor-cognitive actions, providing another means to differentiate the neurophysiological activations associated with cooperative and competitive interpersonal interaction from those inherent to performing a movement-based task.

Another novelty of our study protocol with respect to most joint action studies, regards the assessment of psycho-social factors, e.g., personality traits and team mental models of the players. Specifically, their individual social preferences and motivations and traits of extraversion and introversion can be used as regressors for the group analysis supporting the interpretation of the multilevel analytical results. This approach might help improve and help to explain differences in low and high-performing groups.

We expect that the richness of data recorded with our proposed multimodal experimental setup and study protocol will contribute to the understanding of the neurophysiological mechanisms sustaining daily life joint actions, hence human social behavior. At present, appropriate solutions for the multimodal and multilevel investigation of joint action are lacking in the market, strongly limiting the expansion of these technologies to new fields. Bringing neuroscience and its methodology closer to real-life scenarios has the potential to improve specific social dynamics that characterize various contexts, including workplaces, schools, academia, and sports environments. Some relevant examples of social dynamics in our society include leader-follower relationship, mobbing, bullying, teamwork efficiency, and social inclusion. For instance, outcomes from joint action studies based on our multimodal and multilevel protocol could help designing systems capable of predicting cooperative or competitive behaviors before being overtly expressed. In real-life contexts, performance depends on the ability to understand the other’s intention, which often relies on non-verbal bodily communication and on a balance between cooperation and competition among co-actors. The exploitation of our results and conclusions in organizations and work settings where social behavior is a main feature may provide employers, managers, team leaders, teachers, and educators with knowledge and methodology to anticipate and mitigate conflict situations, improve teamwork efficiency and productivity, and foster learning processes. Individual and group satisfaction and enjoyment derived from teamwork could thus increase, with great benefit for all actors and the society.

## 4 Ethics and dissemination

Our study protocol was designed within a project funded by the European Commission. The general objective is to investigate the neurophysiological underpinnings of human joint action during the performance of natural interactions, that typically occur face-to-face, imply free real-time and real-space coordinated movements and can be both cooperative and competitive. To this aim, the project partners have developed the described new hardware and software solutions for the multimodal recording and multilevel analysis of joint action data, as well as a novel study protocol (described in this article) to fully exploit the novel technological solutions.

The consortium is composed of academic and industrial partners, who carefully address all ethical, legal, social, and safety issues, using the highest standards in Good Laboratory and Good Manufacturing Practices and Ethics applied in European studies. We adhere to all relevant national, European, and international regulations, which conform with the fundamental principles of respect for the physical and moral wellbeing of individuals and ensure freedom in research.

### 4.1 Ethics

The observational studies will be performed in full compliance with European, national and local ethical guidelines, most importantly the Nuremberg Code (1947), the Declaration of Helsinki (1975), and the “Convention on Human Rights and Biomedicine” (Council of Europe, 1997).

Our study was approved by the local Ethics Committees at the respective consortium partners. All volunteers in the study have signed or will sign a written informed consent—integrated with an information sheet—before participating in the study.

#### 4.1.1 Data collection and protection

Appropriate measures will be taken to ensure that data protection, confidentiality and anonymity are maintained. According to the European Legal Framework and in compliance with H2020 ethical guidelines and the EU Regulation 679/2016, personal data will be protected and pseudonymized using a two-level data protection approach. All data will be stored in encrypted and password locked files, whilst transmission of information via electronic means will be performed using encrypted data files and a secure multilevel access system ensuring that only verified users with appropriate rights have access to the data. Steps will be taken to ensure that no individual can be identified in any scientific publication.

#### 4.1.2 Ethical implications of research results

No parts of methods/technology applied to volunteers can be assumed to pose any health risks to the volunteers. The foreseen motion tracking and bioelectrical devices are completely non-invasive and do not expose the volunteers to health risks. Participation in the observational studies will not affect other activities of the volunteers. All devices need to comply with the appropriate legislation related to safety of devices connected to human volunteers. Safety reporting on (serious) adverse events during the observational studies will conform to European and national regulations.

#### 4.1.3 Incidental findings

If any abnormal or unexpected findings are detected during the analysis of the neurophysiological and kinematic data collected, these findings will be notified to the clinician managing the volunteer(s).

### 4.2 Dissemination

Several dissemination activities are planned to increase the international visibility of the consortium and the project, to promote an active knowledge exchange with the scientific community, and to address different scientific communities and industrial leaders. The main activities include:

1. Publication of research results in open-access international peer-reviewed journals. This is the main means for an effective dissemination toward researchers in basic investigations, biomedical engineers, neuroscientists, cognitive psychologists, sport psychologists, and economists.
2. Attendance at international conferences. The most important results will be presented at international conferences that have become main events for diffusing new results on (e.g.) biomedical engineering, EEG, social neuroscience, sport psychology.
3. Organization of international summer schools. Junior researchers can be targeted by organizing summer schools on the interdisciplinary topics relevant for the multimodal and multi-level investigation of joint action.
4. Attendance at international engineering fairs. This will allow us to target industrial partners and stakeholders interested in novel systems for studying joint action with a multimodal approach.
5. Setup and maintenance of the project website, where all activities and main research results are reported, as well as scientific publications, network events, research databases, and a blog, akin to the idea of Horizon 2020 on Open Research Data.
6. Participation in international research, training and industrial networks. All project partners will use their international networks of collaborations to disseminate the research results to their peers.

Communication activities are also planned to target the general public. They include the use of communication materials, e-newsletters, web-based documents, video clips, blogs on social media, public talks and interviews given at local, regional and national TV news and/or scientific programs, Open days at Universities, Researchers’ Night, Pint of Science and Brain Awareness Week, Meetings at Professional Associations and Twitter conferences.

## Ethics statement

The studies involving humans were approved by the Comitato Etico delle Province di Chieti e Pescara (Italy). The studies were conducted in accordance with the local legislation and institutional requirements. The participants provided their written informed consent to participate in this study.

## Author contributions

GT: Conceptualization, Methodology, Writing – review and editing, Data curation, Formal analysis, Investigation, Software, Visualization. PF: Conceptualization, Data curation, Investigation, Methodology, Visualization, Writing – review and editing, Funding acquisition, Writing – original draft. AD: Conceptualization, Data curation, Investigation, Methodology, Writing – review and editing, Formal analysis. KR: Investigation, Writing – review and editing, Software. MK: Investigation, Software, Writing – review and editing. LV: Software, Writing – review and editing, Conceptualization. RB: Conceptualization, Software, Writing – review and editing. HO: Conceptualization, Writing – review and editing. MB: Conceptualization, Writing – review and editing. EF: Conceptualization, Writing – review and editing. FZ: Conceptualization, Writing – review and editing, Investigation, Methodology, Project administration. SC: Conceptualization, Methodology, Project administration, Writing – review and editing, Funding acquisition, Resources, Supervision, Writing – original draft.
